# Effect of Transcranial Magnetic Stimulation (TMS) on Parietal and Premotor Cortex during Planning of Reaching Movements

**DOI:** 10.1371/journal.pone.0004621

**Published:** 2009-02-27

**Authors:** Pierpaolo Busan, Claudia Barbera, Mauro Semenic, Fabrizio Monti, Gilberto Pizzolato, Giovanna Pelamatti, Piero Paolo Battaglini

**Affiliations:** 1 BRAIN Center for Neuroscience, Department of Life Sciences, University of Trieste, Trieste, Italy; 2 Department of Psychology, University of Trieste, Trieste, Italy; 3 Department of Clinical and Experimental Medicine and Clinical and Experimental Neuroscience, University of Trieste, Trieste, Italy; National Institutes of Health, United States of America

## Abstract

**Background:**

Cerebral activation during planning of reaching movements occurs both in the superior parietal lobule (SPL) and premotor cortex (PM), and their activation seems to take place in parallel.

**Methodology:**

The activation of the SPL and PM has been investigated using transcranial magnetic stimulation (TMS) during planning of reaching movements under visual guidance.

**Principal Findings:**

A facilitory effect was found when TMS was delivered on the parietal cortex at about half of the time from sight of the target to hand movement, independently of target location in space. Furthermore, at the same stimulation time, a similar facilitory effect was found in PM, which is probably related to movement preparation.

**Conclusions:**

This data contributes to the understanding of cortical dynamics in the parieto-frontal network, and suggests that it is possible to interfere with the planning of reaching movements at different cortical points within a particular time window. Since similar effects may be produced at similar times on both the SPL and PM, parallel processing of visuomotor information is likely to take place in these regions.

## Introduction

In both the monkey and human brains, it is now well established that two separate streams for processing of visual information exist: a ventral processing stream mainly used for object recognition and a dorsal one mainly involved in processing spatial information needed to perform actions [1]. The dorsal stream involves the superior parietal lobule (SPL) from where information proceeds to the premotor cortex (PM). The SPL is extremely well placed to collect visuospatial information, and transform it into motion [2]. Furthermore, there is strong evidence that the SPL is involved both before and during the production of reaching movements [3]–[5]. Several separations have been proposed to exist in the dorsal stream, all of which suggest that the medial most parieto-frontal region is primarily concerned with the production of reaching movements [6]–[8].

The SPL has strong and largely segregated connections with the dorsal premotor cortex (PMd) [7], [9], [10], where muscles in the arm and hand are represented, which are needed for reaching movements. Moreover, the PMd is directly and indirectly connected to parieto-occipital areas such as area V6A and the medial intraparietal area (MIP), thus allowing a bidirectional flow of information in the dorsal stream [7], [11], [12]. Indeed, it would appear that each parietal area is connected to one or more premotor areas, while each premotor area is mainly in connection with only one parietal area. The cortical areas that are connected usually show strong functional similarities [7].

Contrary to the idea of a stream which flows from one region to another, visuomotor processing may occur simultaneously in a number of highly distributed frontal and parietal areas with similar timings of activation. In fact, it has been suggested that the transformation of visuomotor coordinates takes place at the same time in a set of widely distributed cortical areas [11], and different activations within parieto-frontal circuits may take place concurrently [11], [13], [14] supporting the existence of a parallel processing of visuospatial information [15], [16]. Moreover, in both human and monkeys, the activation time of frontal and parietal areas seems to overlap, confirming the assumption that information is processed in parallel [17]. It should be mentioned, however, that a hierarchy of analytic steps has been already suggested [18] and evidence of sequential activation from parietal to frontal areas has been found [10], [19], [20].

In spite of the large amount of work dedicated to this subject, parallel and/or serial processing still remains poorly understood. In the present study, the contribution of parieto-frontal areas to the dorsal stream of visuomotor processing was investigated. Specifically, the superior parietal and dorsal premotor cortex were mapped by interfering with their activity using single pulse transcranial magnetic stimulation (TMS) during planning of reaching movements within particular time windows, and observing if different effects related to the point of stimulation and/or location of target in peripersonal space exist.

TMS can inhibit or facilitate information processing in stimulated brain areas during the execution of a task, provided that those areas are actually involved [21], [22], [23]. Normally, it can penetrate almost 2 cm under the stimulation site, thus reaching the cerebral cortex, when the intensity of stimulation is adjusted to 120% of resting motor threshold [24]. Furthermore, the radius of the electric field induced by a 10–15 cm figure-8 coil extends by no more than 1.5 cm at the same stimulation intensity [25], therefore allowing delivery of discrete stimulations with the possibility of obtaining different results from neighbouring regions.

## Results

Mean reaction time (m-RT) was a crucial measure as it determined the timing of TMS delivery, and showed both inter- and intra-subject variability depending on skill improvement. For this reason, not all values were presented, but ranged from 482.99±51.8 ms in the fastest subject to 765.65±75.8 ms in the slowest with a mean value of 631.71±92.4 ms between experiments. Three experiments were conducted in the parietal cortex and three in the premotor dorsal cortex. Statistical analysis of reaction time (RT) showed significant results in only two experiments; the other experiments will not be discussed, and only the recorded mean RT are reported in Table 1 and 2. Analysis of movement times did not show statistically significant differences.

**Table 1:** Mean reaction times and standard deviations (in ms) collected in the TMS and NO-TMS experiments in the parietal cortex.

pts	target location	TMS at 50% of m-RT		TMS at 75% of m-RT		TMS at 90% of m-RT	
		TMS	NO-TMS	TMS	NO-TMS	TMS	NO-TMS
**a**	central	652.1±133.4	672.4±135.7	641.3±91.7	623.1±103.5	602.3±44.4	615. 4±43.1
	left	676.1±137.1	720.7±147.1	657.4±103.3	664.9±114.1	619.1±50.9	624.4±66.6
	right	662.5±138.2	679.1±123.6	619.1±75.1	619.1±78.4	596.1±61.6	597.1±37.4
**b**	central	648.2±125.9	637.3±107.5	649.9±89.7	642. 5±83.9	624.3±49.6	619.5±52.9
	left	658.5±122.9	668.2±112.1	673.3±105.1	680.1±95.6	648.3±77.1	648.7±81.2
	right	645.7±114.6	656.6±124.9	617.1±74.1	612.8±91.1	622.6±59.5	620.0±52.1
**c**	central	654.5±143.7	662.8±140.2	624.0±127.8	624.6±117.1	607.1±43.9	595.3±44.7
	left	670.0±163.9	700.7±156.4	658.5±97.3	687.7±122.4	623.1±54.6	617.3±57.5
	right	629.8±141.5	659.9±142.6	610.9±92.9	635.±118.9	594.0±44.7	600.2±55.6
**d**	central	649.1±127.5	647.7±112.3	***595.4***±***72.7***	***637.0***±***82.2***	630.6±56.6	632.8±54.1
	left	659.9±145.1	684.4±150.7	***644.9***±***85.1***	***652.3***±***89.8***	628.7±58.4	643.8±60.6
	right	635.1±108.9	646.5±115.5	***602.1***±***80.5***	***613.3***±***92.1***	623.2±38.2	615.1±37.8
**e**	central	627.5±101.4	649.5±131.1	630.2±88.8	598.9±86.5	612.6±41.9	622.6±46.1
	left	654.2±114.9	670.9±122.1	648.0±92.2	664.5±115.5	645.8±43.1	628.7±46.8
	right	627.0±124.5	652.2±119.9	604.2±79.7	593.6±57.2	623.5±45.8	599.2±30.8

Data are reported for each of the three timing conditions, relatively to points of stimulation and target location. Bold characters indicate statistically significant comparisons.

**Table 2:** Mean reaction times and standard deviations (in ms) collected in the TMS and NO-TMS experiments in the premotor dorsal cortex.

pts	target location	TMS at 50% of m-RT		TMS at 75% of m-RT		TMS at 90% of m-RT	
		TMS	NO-TMS	TMS	NO-TMS	TMS	NO-TMS
**f**	central	624.4±99.2	640.5±96.8	615.6±89.7	610.1±81.1	632.5±133.1	614.8±121.3
	left	668.5±114.7	665.7±91.9	632.7±97.9	648.7±87.6	636.2±141.6	638.9±180.1
	right	631.1±97.2	630.9±87.7	606.6±54.9	616.5±81.9	618.1±146.6	626.3±151.8
**g**	central	645.9±112.7	628.9±86.5	571.3±97.2	585.2±99.2	644.5±131.1	639.5±160.7
	left	667.2±134.2	717.1±127.6	625.3±84.8	633.1±121.3	587.5±123.6	582.9±164.1
	right	626.7±95.5	657.6±92.6	585.4±97.9	603.8±101.2	633.2±129.5	642.6±129.7
**h**	central	598.4±116.9	624.0±95.1	***570.8***±***77.4***	***596.1***±***74.7***	620.2±139.5	593.2±130.9
	left	642.5±151.1	685.2±142.1	***615.5***±***112.1***	***661.4***±***105.6***	633.8±147.4	625.3±144.8
	right	607.6±118.4	645.3±111.2	***588.5***±***78.3***	***608.8***±***108.8***	599.9±124.5	618.2±152.4
**h1**	central	N/A	N/A	541.7±83.5	528.5±69.4	N/A	N/A
	left	N/A	N/A	547.9±93.1	542.6±103.6	N/A	N/A
	right	N/A	N/A	532.1±86.7	544.6±101.4	N/A	N/A
**i**	central	637.3±111.2	619.5±93.9	590.9±108.6	576.4±65.4	613.6±123.1	593.6±138.2
	left	659.1±125.7	695.5±103.7	627.2±80.5	638.5±108.6	598.7±138.7	605.6±168.1
	right	624.8±108.7	640.8±73.5	579.2±95.7	578.1±100.6	627.1±149.8	614.1±139.5
**j**	central	627.2±128.6	649.2±120.3	606.1±79.1	616.6±119.3	601.8±107.2	605.5±111.4
	left	660.9±156.5	713.4±145.2	653.9±81.9	643.6±72.4	648.4±112.3	640.3±118.9
	right	626.7±132.1	639.5±103.5	608.3±76.8	619.2±74.1	646.8±111.6	622.3±135.6

Data are reported for each of the three timing conditions, relatively to points of stimulation and target location. Bold characters indicate statistically significant comparisons.

### TMS at 75% of m-RT in parietal cortex (experiment 2)

**Figure 1:**
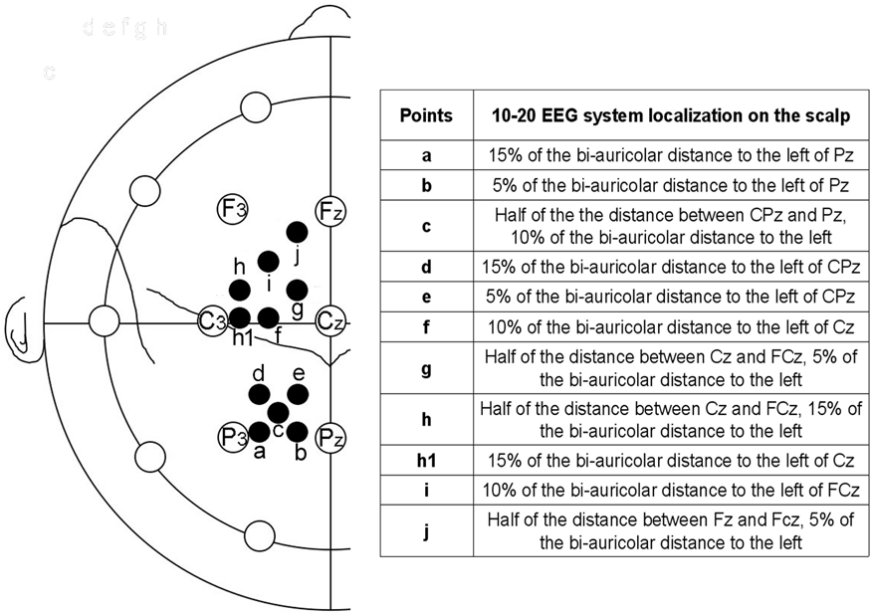
representation of the stimulated points where locations on a model are indicated.

This experiment involved 11 right-handed subjects (5 men and 6 women, range 22–41 yr, mean age 25.64±5.84). A statistically significant interaction was found only between TMS and position of the stimulation point on the scalp (F_4,40_ = 3.411; p = 0.017). The mean RT was significantly shorter in the TMS vs. no-TMS condition only in a more rostral and lateral scalp location (point d in Fig. 1; t = 3.416; p = 0.0066).

**Figure 2:**
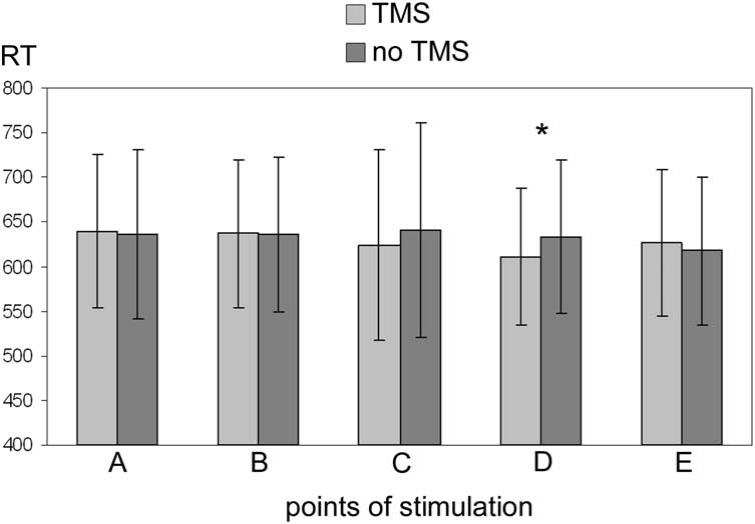
reaction times (in ms) obtained in the parietal cortex. Data are grouped for all three object locations for each point of stimulation with or without TMS. Mean values and standard deviations are reported. The asterisk indicates the only statistically significant difference.

Table 1 and Figure 2 show the data regarding the mean RT for each stimulated point with and without TMS pulse, grouped for all three object locations. Thus, the TMS effect can be clearly localized on the scalp and appears to affect all three points in space.

### TMS at 75% of m-RT in dorsal premotor and motor cortex (experiment 5)

This experiment involved 8 right-handed subjects (2 men and 6 women, range 20–52, mean age 25.7±10.03). The results showed an interaction only between TMS and location of stimulation on the scalp (F_5_,_35_ = 2.651; p = 0.039). Moreover, post-hoc analysis showed that TMS significantly decreased reaction times in a specific point of the dorsal premotor cortex (point h in Fig. 1; t = 5.575; p = 0.0003).

This location was situated about 2 cm rostral to the representation of hand muscles in the primary motor cortex, as previously determined when stimulating first dorsal interosseous (FDI) muscle to determine motor threshold. In 5 subjects (5 men; range 21–43, mean age 28.4±8.6), point h was stimulated at 110% of resting motor threshold to investigate the possibility of current diffusion to the primary motor cortex. Motor evoked potentials of hand muscles were reproducibly induced in one subject only, and occasionally in another subject. However, we are confident that the experimental findings for point h are not due to the direct diffusion of TMS to primary motor cortex since point h1, which usually corresponded to the best motor representation for FDI muscle, was actually stimulated at 110% of resting motor threshold, with no significant results (Tab. 2). Point h1 was also stimulated at 90% of resting motor threshold in 5 of the subjects to determine if the present results are related to subthreshold activation of the hand motor cortex. No statistically significant differences were observed (TMS: 530.4±78.9 ms, no-TMS: 527.7±78.6 ms; t = 0.949, p = 0.396).

**Figure 3:**
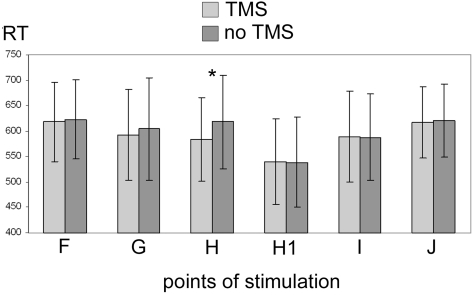
reaction times (in ms) obtained in dorsal premotor cortex. Data are grouped for all three object locations for each point of stimulation with or without TMS. Mean values and standard deviations are reported. The asterisk indicates the only statistically significant difference.

Table 2 and Figure 3 show the data regarding the mean RT for each stimulated point with and without TMS pulse, grouped for all three object locations.

### No-Reaching experiment

In the first control experiment, TMS was applied to 10 subjects (6 men and 4 women, range 19–52, mean age 25.2±9.6) on the effective cortical locations (points d and h in Fig. 1) at the corresponding effective time of stimulation (75% of m-RT). Statistical comparisons revealed significant differences between TMS and no-TMS conditions only when point h was stimulated (point d: TMS = 673.9±76.1 ms, no-TMS = 678.7±79.2 ms; t = 0.304, p = 0.768; point h: TMS = 675.6±73.9 ms; no-TMS = 697.7±73.9 ms; t = 2.556, p = 0.031).

### No auditory cue experiment

In the second control experiment, TMS was applied to 6 subjects (4 men and 2 women, range 21–56, mean age 30.3±14.1) on the effective scalp locations (points d and h in Fig. 1) at the corresponding time of stimulation (75% of m-RT). In the normal experiments, half of the reaction time was usually needed to open the eyes and the other half to reach the target. In order to validate the effect of TMS delivery in this “long” reaction time, a series of control experiments was performed in the light with eyes open. Under these conditions, the reaction time almost halved and TMS was applied at 50% of subjects' medium reaction time to obtain a timing correspondence with the original experiments. Statistical comparisons revealed significant differences between TMS and no-TMS conditions when both point d and point h were stimulated (point d: TMS = 357.8±79.9 ms, no-TMS = 369.3±84.3 ms; z = −2.201, p = 0.031; point h: TMS = 347.9±63.0 ms; no-TMS = 360.5±63.3 ms; t = 4.349, p = 0.007).

### Anatomical localization

**Figure 4:**
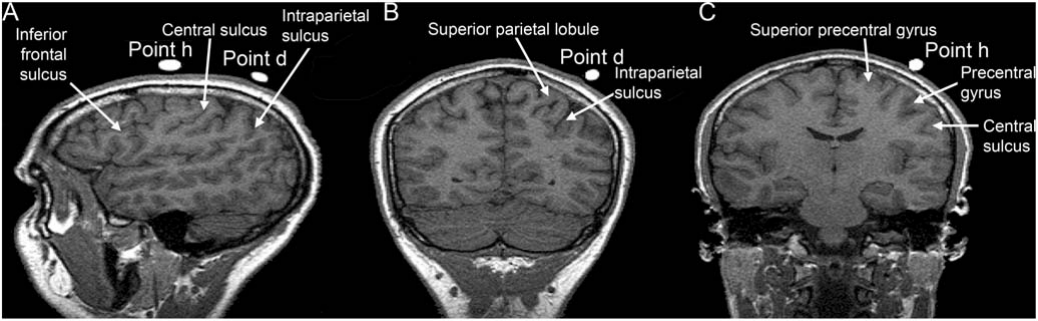
location of effective points of stimulation on anatomical magnetic resonance scanning of one subject, revealed by vitamin E pills. A: sagittal section (point h anterior and point d posterior); B, C: coronal section through point d (parietal lobe) and point h (frontal lobe), respectively. Anatomical locations were identified according to Duvernoy [26].

In one subject, vitamin E pills were positioned over scalp locations where statistically significant reductions in RT were observed (points d and h in Fig. 1). MR imaging (Fig. 4) showed that point d was situated over the parietal lobe in correspondence to the intraparietal sulcus. Due to coil orientation, in this case stimulation probably involved the immediately adjacent part of the superior parietal lobule (Brodmann area 7) rather than the cortex of the intraparietal sulcus or the inferior parietal lobule. Point h was comfortably situated over the premotor dorsal cortex (Brodmann area 6) in a region between the superior precentral sulcus and the precentral gyrus. Taking into account the anatomical variability in parietal regions [27], and also considering a range of ±8 mm when using a 10-20 EEG system to individuate the correspondence between position of electrodes and underlying cortex [28], the parietal experiment stimulation probably involved a region of cortex in SPL, presumably situated nearby the intraparietal sulcus, while premotor stimulation affected the PMd area.

## Discussion

In the present experiments, 4 points were stimulated in PMd (points g–j in Fig. 1), 2 in the primary motor cortex (points f and h1 in Fig. 1) and 5 in the parietal cortex (points a–e in Fig. 1). These regions were identified using the 10-20 EEG coordinate system [28], [29]. Sulcal and gyral anatomy was also evaluated in one subject using MR (Fig. 4). Moreover, motor responses during TMS stimulation were usually experienced by subjects when the coil was positioned on point h1 (hand), and were occasionally reported when the coil was positioned on point f (shoulder), thus functionally confirming their location on the primary motor cortex.

The distance between points to be stimulated was as short as possible so that conspicuous regions of cortex would remain unaffected and overlap between neighbouring regions could be avoided. In fact, the closest distance between adjacent points was about 3.5 cm, depending upon the cranial dimension. An exception was made for control point h1, normally corresponding to motor representation of hand muscles, which was about 2 cm away from points h and f (see Fig. 1). Moreover, the radius of the electric field induced by TMS is known to be about 1.5 cm when it is applied at 120% of resting motor threshold [24]. As a consequence, the periphery of the electric field at any particular point could not directly influence the adjacent ones, and thus the observed effects were specific for the stimulated point.

The TMS effect on the parietal cortex was related to the planning of reaching movements and not to non-specific visual, motor or attentional influence, since no effect was observed in the no-reaching experiment, where location of visual targets and motor signals were however present, but the movement was not a reaching one. On the other hand, the effect in the premotor cortex was replicated in the no-reaching experiment, suggesting that it could be related to motor programming [30] and/or start of movement rather than to only planning of reaching. Sham stimulation was not used because the effect was limited to specific scalp positions, so that all the other stimulated points may be considered as sham stimulations. Thus, the present results are not likely to be due to a TMS-induced intersensory facilitation [31].

Finally, control experiments showed that the results obtained for point h are not related to diffusion of current to the primary motor cortex. Moreover, a possible confounding factor of the experiment, i.e. variability in reaction times of eye-opening after the go-signal, was not relevant to the task. In fact, the time-windows of effective stimulation also remained unchanged after the elimination of the go-signal to eye-opening time interval.

### Parietal cortex

TMS applied at 75% of the m-RT over a specific location in the parietal cortex (point d in Fig. 1) significantly shorted the reaction time independently of the target position in space. It is generally accepted that TMS induces a virtual lesion in the affected region [32], but facilitatory effects of single-pulse magnetic stimulation have also been described in different cognitive fields, ranging from picture naming to motion perception [22], [33]. It has been suggested that stimulating an inactive region of cortex involved in a specific task could lead to subsequent facilitation in the observed behaviour [23]. In fact, a TMS state-dependent effect has been proposed to explain the facilitatory and inhibitory effects induced by TMS. When TMS is applied before the onset of a cognitive process, all neural populations are at a baseline level of activity and are thus facilitated to a similar extent [23]. This results in a general increase in cortical excitability, reflected in an enhanced sensitivity to subsequent sensory stimulation [23], [34], [35]. On the other hand, when TMS is applied during a cognitive process, an activity lack of balance exists in the stimulated region: neurons not involved in the process are less active as opposed to neurons that are critical to the cognitive function under investigation [23]. Because of this activity imbalance, attributes encoded by all neural populations are not equally facilitated. Rather, TMS preferentially facilitates attributes encoded by neurons that are not involved in the cognitive process (as these neurons are relatively inactive) with respect to the highly active neurons that are critical to the cognitive function under investigation. This effectively reduces the signal-to-noise ratio and produces behavioural disruption [23].

Referring to the present results, the observed facilitatory effects might be due to pre-activation of the stimulated area a few milliseconds before its normal activation, thus speeding up the start of the reaching movement [23].

The parieto-occipital cortex of both monkeys and humans is easily activated by peripheral visual stimulation related to planning of reaching [36]–[41], while more anterior fronto-parietal regions mainly respond to reaching with foveal vision [40], [42]. The present data confirm the role played by the parietal cortex in the planning of reaching movements, but the entire visual field was independently affected by TMS. In fact, both central and peripheral vision were equally involved, suggesting that these anterior parietal regions could be involved in both central and peripheral visuomotor integration.

### Dorsal premotor cortex

The reaction time after TMS over PMd (point h in Fig. 1) was significantly shortened at a stimulation time of 75% of m-RT, and this effect was evident for all target positions. As for parietal location, the TMS effect might be due to pre-activation of the stimulated area before its normal activation [23], thus speeding up the start of the reaching movement. This area is presumed to be involved in prosecution of the dorsal stream towards the motor cortex and in the processing of reaching movements [9], [10], [13], [14], [43]. It has also been suggested that the principal role of PMd could be to activate selected reaching actions [44], but since almost no simultaneous activation occurs in the parietal cortex (present experiments), it may be that PMd also plays a role in the preparation of reaching movements.

### Conclusions

The results of this study suggest that localized and concomitant cortical facilitation could be evoked with TMS during planning of reaching movements in humans, supporting the presence of a parallel flow of activation in the dorsal stream and in its prosecution. This topic is still debated, but it has been suggested that in humans the superior parietal lobule and premotor cortex could be activated sequentially rather than simultaneously during movement preparation and/or execution [10], [19], [20]. On the other hand, an fMRI study [45] also proposed that pointing with central gaze activates a diffuse fronto-parietal network, comprising the areas thought to be involved in the present study. However, no information about temporal involvement of areas was provided in that study. More recently, localized and concomitant cortical activations in parietal lobe and premotor cortex have also been reported in an electroencephalographic study on planning of pointing movements in humans [17], indicating reverberant and coincident activation of the fronto-parietal cortex. Finally, in a number of studies in monkeys, overlapping and concomitant activation of different areas in the parietal lobe and premotor cortex during planning of reaching movements have been clearly demonstrated [11], [13], [14].

TMS applied at 50% and 90% of m-RT did not evoke any effect. Following the present interpretation, 50% of m-RT might have been too early and 90% too late with respect to the actual activation of the parietal and premotor cortices. Indeed, 50% and 90% of m-RT preceded and followed the time of effective stimulation by about 100 ms, respectively.

Finally, several different visuomotor parietofrontal circuits have been proposed to exist [7], [9], with each primarily involved in a specific task, and only one of these parieto-frontal circuits might have been stimulated in the present experiments. Indeed, not all points in the parietal and premotor cortices were affected, leaving the possibility that they might be involved in different tasks.

## Materials and Methods

The present study involved 76 healthy volunteers (range 19–56, mean age 27.1±8.0), who were allocated to six sets of experiments, in addition to two control experiments. All individuals were right-handed according to the Oldfield test [46]. Subjects were provided with information regarding TMS and any related risks before signing an informed consent agreement in accordance with the Declaration of Helsinki. Subjects could leave the experiment at any time, although all completed the experimental sessions. Permission from the Ethics Committee of the University of Trieste was also obtained. During experiments, each subject was comfortably seated in front of a table, and was asked to place his right hand on a light detector that was fixed in a hole on the table, just in front of the subject's chest. A cross, drawn on the table at 35 cm in front of the subject along the midline, was used to maintain steady fixation during the experiment. A small metal cylinder was placed on the cross or at 40° from it, on either the right or left. The cylinder was connected to an impedance meter which allowed measurement of the time elapsed between the start of the movement (signalled by the light sensor) and its completion (signalled by touching the cylinder). A digital video camera (Sony DCR-SR30E, sampling rate 25 Hz) was placed in front of the subject to record arm and eye movements, and to discard incorrectly performed trials. At the beginning of each trial, subjects were asked to close their eyes. Since complete darkness is difficult to obtain, experiments were performed in a normally illuminated environment. To avoid visual distraction before initiating the task, subjects were asked to start each trial with their eyes closed, so as to permit an on-line planning of reaching movements at the go-signal. An auditory tone signalled subjects to open their eyes, and to reach and touch the target cylinder, as soon as possible, while maintaining steady fixation on the cross, independently from the position of the cylinder itself. All events were under control of a PCMCIA acquisition board (NI-DAQ 6024E, National Instruments, Texas, USA) connected to LABVIEW PC software, which instructed the experimenter where randomly place the cylinder, and recorded both reaction and movement time (RT and MT), which were measured as the time elapsed between the tone and start of hand movement (RT) and as the duration of hand movement (MT), respectively.

Single TMS pulses (Medtronic MagPro R30) were delivered on the skull at different points determined according to the 10-20 EEG coordinate system [28], [29] and marked with stickers on a Lycra cap. This allowed a localization error with a mean standard deviation of the presumed underlying cortical points of ±8 mm [28]. We performed six sets of experiments: three in the premotor cortex and three in the parietal one. In the premotor experiments, four points in the dorsal premotor area and two in the primary motor area were stimulated. In the parietal experiments, five points in the parietal cortex were stimulated (Fig. 1). Point h1 usually corresponded to the optimal representation of the first dorsal interosseal muscle (FDI), and was stimulated in only one set of experiments, as a control point. In each subject, points were always stimulated in a different order.

A seven cm figure-8 coil (Medtronic C-B60) was used for TMS delivery (pulse duration: 280 µs). The coil was positioned and secured in place on the scalp by fixing it to a mechanical arm. Its position was continuously checked and readjusted by the experimenter when necessary. The coil handle was always oriented backwards and parallel to the midline in the premotor experiments, and 45° backwards, toward the subject's right side, in the parietal experiments.

In each subject, the motor threshold was preliminarily determined as the best cortical point activating the FDI muscle, as determined by EMG, measured as the stimulus intensity triggering at least a 50 µV response in 50% of 10 consecutive stimulations. The stimulation intensity of the TMS pulse was then set at 120% of the FDI motor threshold in the parietal experiments, and at 110% in the premotor experiments to reduce the direct diffusion of stimulation to the primary motor cortex.

Before starting the experiment, subjects were required to perform a series of 20 reaching trials with the target randomly distributed at the centre, right or left in order to measure their mean reaction time (m-RT), which was then used to apply the TMS at the right time after the go-signal. Pulses were applied at 50% of m-RT (corresponding to experiment 1 in the parietal cortex and experiment 4 in the premotor cortex), 75% of m-RT (corresponding to experiment 2 in the parietal cortex and experiment 5 in the premotor cortex) and 90% of m-RT (corresponding to experiment 3 in the parietal cortex and experiment 6 in the premotor cortex). In each subject, only one of the three TMS stimulation times and one of the two cortical regions was studied in order to prevent fatigue and avoid extended exposure to magnetic stimulation. Stimulation times were intended to ensure that the magnetic stimulus reached subjects when their eyes were steadily opened following the loudspeaker signal. TMS at 50% of m-RT, however, was delivered around the time of eye-opening. This means that although TMS delivery was coincident with eye-opening, it was sometimes either “late” or “too early”. If after a series of trials subjects reduced their RT by more than 20% as a consequence of improved skills, a new m-RT was measured and the timing of TMS delivery was subsequently modified. Reaction times were considerably long because of the reaction time paradigm, where the time elapsed from the acoustic signal to arm movement (reaction time) comprised both the time required to open their eyes and that needed to move towards the target. Subjects performed 42 consecutive randomized trials on each stimulated point: 21 with TMS and 21 without TMS. Of each set, 7 had the target on the right, 7 on the left and 7 in the centre. As already mentioned, all data regarding timing were controlled and collected automatically using a PCMCIA acquisition board (NI-DAQ 6024E, National Instruments, Texas, USA) controlled by a LABVIEW PC software.

Two control experiments were also performed. In the first, subjects had to move their thumb instead of their arm. The thumb was moved away from the light sensor whilst the target was in the central position, and it was not moved when the target was to the left or to the right. In this way, the reaching component of the task was eliminated, maintaining visual detection, attention and motor planning. Subjects performed a total of 24 consecutive reaching movements for each stimulation point on the scalp: 12 toward the centre, 6 toward the left and 6 toward the right. TMS was randomly delivered in half of the trials.

In the second control experiment, points at the corresponding effective times of stimulation were further investigated. The auditory cue and the reaction time related to eye-opening were eliminated from the task in order to control for the precision of TMS delivery during planning of reaching movements. A target-light appeared randomly on the table in one of the three locations used for the original experiments (centre, left and right), signalling the start of the trial. For each point of stimulation, 30 reaching movements were executed: 10 towards the centre, 10 towards the left and 10 towards the right. TMS was randomly delivered in half of the trials.

### Anatomical localization

To corroborate the correspondence between points of stimulation on the scalp and underlying cortical areas, based on the 10-20 EEG system [28], [29] one subject underwent T1-weighted anatomical magnetic resonance (MR) scanning (slide thickness 1.5 mm, TR 10.26 ms, ET 4.6 ms, fov 240×240, acquisition matrix 256×256 pixels, pixel spacing 0.93×0.93). Points of interest were marked with vitamin E pills.

### Statistical analysis

TV recordings were analyzed off-line and all trials where eyes did not remain still on the central fixation cross for the entire duration of the trial were excluded. To avoid the influence of inadequate attention on movement performance, all trials with a reaction time longer than 1000 ms or shorter than 100 ms and/or movement time longer than 700 ms or shorter than 100 ms were also excluded. Moreover, incorrectly performed reaching movements were also discarded, i.e. when the reaction to acoustic signal was not prompted, when subjects corrected their trajectory or when they hesitated after initiating hand movement. About 10% of the registered data was eliminated due to discrepancies in reaction times.

As data were normally distributed (Shapiro-Wilk test), no transformation or correction on reaction or movement times was needed, and analysis was conducted directly on row values. Moreover, homogeneity of variance (Test F) was successfully checked between single conditions in every experiment. The only data that was not normally distributed was that for the parietal cortex in the second control experiment. In this case, a Wilcoxon signed rank test was used for analysis.

Analysis was conducted with ANOVA. Specifically, a three-way ANOVA (significance level set at p<0.05) considering the main effects and interactions between TMS conditions, location of stimulation on the scalp and target position (centre, left and right) was conducted. If interactions were statistically significant, post-hoc analysis was performed. If significant interaction was found only between two main factors, a paired Student's t-test was done (significance level set at p<0.05, Bonferroni corrected). If significant interaction was present for all three main factors, a two-way ANOVA was conducted on each pair of parameters. If significance was still present, a paired Student's T-Test was used (significance level set at p<0.05, Bonferroni corrected).

## References

[1] Milner AD, Goodale MA (2006). The Visual Brain in Action. 2nd Edition.

[2] Rizzolatti G, Fogassi L, Gallese V (1997). Parietal cortex: from sight to action.. Curr Opin Neurobiol.

[3] Snyder LH, Batista AP, Andersen RA (1997). Coding of intention in the posterior parietal cortex.. Nature.

[4] Cui H, Andersen RA (2007). Posterior parietal cortex encodes autonomously selected motor plans.. Neuron.

[5] Buneo CA, Batista AP, Jarvis MR, Andersen RA (2008). Time-invariant reference frames fro parietal reach activity.. Exp Brain Res.

[6] Tannè J, Boussaud D, Boyer-Zeller N, Rouiller EM (1995). Direct visual pathways for reaching movements in the macaque monkey.. Neuroreport.

[7] Matelli M, Luppino G (2001). Parietofrontal circuits for action and space perception in the macaque monkey.. Neuroimage.

[8] Galletti C, Kutz DF, Gamberini M, Breveglieri R, Fattori P (2003). Role of the medial parieto-occipital cortex in the control of reaching and grasping movements.. Exp Brain Res.

[9] Tannè-Gariepy J, Rouiller EM, Boussaud D (2002). Parietal inputs to dorsal versus ventral premotor areas in the macaque monkey: evidence for largely segregated visuomotor pathways.. Exp Brain Res.

[10] Davare M, Andrei M, Cosnard G, Thonnard JL, Olivier E (2006). Dissociating the role of ventral and dorsal premotor cortex in precision grasping.. J Neurosci.

[11] Caminiti R, Ferraina S, Battaglia-Mayer A (1998). Visuomotor transformations: early cortical mechanisms of reaching.. Curr Opin Neurobiol.

[12] Johnson SH, Grafton ST (2003). From “acting on” to “acting with”: the functional anatomy of object-oriented action schemata.. Prog Brain Res.

[13] Battaglia-Mayer A, Ferraina S, Marconi B, Bullis JB, Lacquaniti F (1998). Early motor influences on visuomotor transformations for reaching: a positive image of optic ataxia.. Exp Brain Res.

[14] Marconi B, Genovesio A, Battaglia-Mayer A, Ferraina S, Squatrito S (2001). Eye-hand coordination during reaching. I Anatomical relationship between parietal and frontal cortex.. Cereb Cortex.

[15] Kalaska JF, Crammond DJ (1992). Cerebral cortical mechanisms of reaching movements.. Science.

[16] Kalaska JF, Sergio LE, Cisek P (1998). Cortical control of whole-arm motor tasks.. Novartis Found Symp.

[17] Naranjo JR, Brovelli A, Longo R, Budai R, Kristeva R (2007). EEG dynamics of the frontoparietal network during reaching preparation in humans.. Neuroimage.

[18] Favilla M, De Cecco E (1996). Parallel direction and extent specification of planar reaching arm movements in humans.. Neuropsychologia.

[19] Schluter ND, Rushworth MF, Passingham RE, Mills KR (1998). Temporary interference in human lateral premotor cortex suggests dominance for the selection of movements. A study using transcranial magnetic stimulation.. Brain.

[20] Koch G, Franca M, Del Olmo MF, Cheeran B, Milton R (2006). Time course of functional connectivity between dorsal premotor and contralateral motor cortex during movement selection.. J Neurosci.

[21] Anand S, Hotson J (2002). Transcranial magnetic stimulation: Neurophysiological applications and safety.. Brain Cogn.

[22] Silvanto J, Cattaneo Z, Battelli L, Pascual-Leone A (2008). Baseline cortical excitability determines whether TMS disrupts or facilitates behavior.. J Neurophysiol.

[23] Silvanto J, Muggleton NG (2008). New light through old windows: moving beyond the “virtual lesion” approach to transcranial magnetic stimulation.. Neuroimage.

[24] Roth Y, Amir A, Levkovitz Y, Zangen A (2007). Three-dimensional distribution of the electric field induced in the brain by transcranial magnetic stimulation using figure-8 and deep H-coils.. Journal Clin Neurophysiol.

[25] Thielscher A, Kammer T (2004). Electric field of two commercial figure-8 coils in tms: calculation of focality and efficiency.. Clin Neurophysiol.

[26] Duvernoy HM (1999). The human brain. Surface, blood supply, and three-dimensional sectional anatomy. 2nd edition.

[27] Ryan S, Bonilha L, Jackson SR (2006). Individual variation in the location of the parietal eye fields: a TMS study.. Exp Brain Res.

[28] Okamoto M, Dan H, Sakamoto K, Takeo K, Shimizu K (2004). Three-dimensional probabilistic anatomical cranio-cerebral correlation via the international 10-20 system oriented for transcranial functional brain mapping.. Neuroimage.

[29] Herwig U, Satrapi P, Schonfeldt-Lecuona C (2003). Using the international 10-20 EEG system for positioning of transcranial magnetic stimulation.. Brain Topogr.

[30] Mars RB, Piekema C, Coles MG, Hulstijn W, Toni I (2007). On the programming and reprogramming of actions.. Cereb Cortex.

[31] Sawaki L, Okita T, Fujiwara M, Mizuno K (1999). Specific and non-specific effects of transcranial magnetic stimulation on simple and go/no-go reaction time.. Exp Brain Res.

[32] Walsh V, Pascual-Leone A (2003). Transcranial magnetic stimulation. A neurochronometrics of mind.

[33] Mottaghy FM, Sparing R, Topper R (2006). Enhancing picture naming with transcranial magnetic stimulation.. Behav Neurol.

[34] Grosbras MH, Paus T (1998). Transcranial magnetic stimulation of rontal eye field facilitates visual awareness.. Eur J Neurosci.

[35] Topper R, Mottaghy FM, Brugmann M, Noth J, Huber W (1998). Facilitation of picture naming by focal transcranial magnetic stimulation of Wernicke's area.. Exp Brain Res.

[36] Galletti C, Fattori P, Kutz DF, Battaglini PP (1997). Arm movement-related neurons in the visual area v6a of the macaque superior parietal lobule.. Eur J Neurosci.

[37] Galletti C, Fattori P, Kutz DF, Gamberoni M (1999). Brain location and visual topography of cortical area v6a in the macaque monkey.. Eur J Neurosci.

[38] Fattori P, Gamberini M, Kutz DF, Galletti C (2001). Arm-reaching neurons in the parietal area V6A of the macaque monkey.. Eur J Neurosci.

[39] Fattori P, Kutz DF, Breveglieri R, Marzocchi N, Galletti C (2005). Spatial tuning of reaching activity in the medial parieto-occipital cortex (area V6A) of macaque monkey.. Eur J Neurosci.

[40] Prado J, Clavagnier S, Otzenberger H, Schelber C, Kennedy H (2005). Two cortical systems for reaching in central and peripheral vision.. Neuron.

[41] Himmelbach M, Karnath HO, Perenin MT, Franz VH, Stockmeier K (2006). A general deficit of the “automatic pilot” with posterior parietal cortex lesion?. Neuropsychologia.

[42] Battaglia-Mayer A, Mascaro M, Caminiti R (2007). Temporal evolution and strenght of neural activity in parietal cortex during eye and hand movements.. Cereb Cortex.

[43] Rizzolatti G, Luppino G, Matelli M (1998). The organization of the cortical motor system: New concepts.. Electroencephalogr Clin Neurophysiol.

[44] Kalaska JF, Scott SH, Cisek P, Sergio LE (1997). Cortical control of reaching movements.. Curr Opin Neurobiol.

[45] Connolly JD, Goodale MA, DeSouza JFX, Menon RS, Vilis T (2000). A comparison of frontoparietal fMRI activation during anti-saccades and anti-ponting.. J Neurophysiol.

[46] Oldfield RC (1971). The assessment and analyisis of handedness: the Edinburgh inventory.. Neuropsychologia.

